# Engineering a vascularized-osteogenic microenvironment to enhance bone regeneration via a 3D-printed composite scaffold with progressive-release bio-factors

**DOI:** 10.1186/s12967-026-08090-5

**Published:** 2026-04-10

**Authors:** Tianao Shao, Guangyu Hu, Yuncheng Bai, Yong Li, Han Xiao, Yaoxian Han, Shuyun Zeng, Yuhui Ma, Lei Zhang

**Affiliations:** 1 State Key Laboratory of Primate Biomedical Research, Institute of Primate Translational Medicine, Kunming University of Science and Technology, Kunming, Yunnan 650500 China; 2 Yunnan Key Laboratory of Primate Biomedical Research, Kunming, Yunnan, 650500 China; 3 Faculty of Medical Science, Kunming University of Science and Technology, Kunming, 650032 China; 4 The First People’s Hospital of Yunnan Province, Affiliated Hospital of Kunming University of Science and Technology, Kunming, 650032 China; 5 First Department of Thoracic Surgery, Yunnan Cancer Hospital, Third Affiliated Hospital of Kunming Medical University, Peking University Cancer Hospital Yunan, Kunming, 650118 China

**Keywords:** Bone defect, Tissue engineering, 3D printing

## Abstract

**Background:**

Critical-sized bone defects remain challenging to repair because successful regeneration requires both mechanical stability and the coordinated promotion of osteogenesis and vascularization. To address these needs, we developed a composite scaffold (GV@PHL) that integrates structural support with sustained pro-angiogenic signaling.

**Methods:**

A 3D-printed framework composed of polycaprolactone (PCL), nano-hydroxyapatite (nano-HA), and Laponite (PHL) was fabricated to form an interconnected porous architecture with intrinsic osteogenic potential and printability. A GelMA hydrogel was photo-crosslinked within the scaffold pores and covalently tethered to vascular endothelial growth factor (VEGF) to create a photo-embedded GelMA–VEGF phase, enabling sustained VEGF release. The composite scaffold (GV@PHL) was evaluated through in vitro and in vivo experiments to assess architecture stability, osteogenic differentiation, VEGF release behavior, endothelial cell responses, and vascularization.

**Results:**

The GV@PHL scaffold maintained a stable porous architecture and exhibited synergistic performance combining structural integrity with biological activity. The PHL framework supported osteogenic differentiation, while the photo-crosslinked GelMA–VEGF hydrogel enabled controlled, sustained release of VEGF. Released VEGF promoted endothelial cell survival and enhanced vascularization in vitro and in vivo, demonstrating coordinated support for osteogenesis and angiogenesis.

**Conclusions:**

GV@PHL represents a practical strategy for integrating structural design and biological function in bone tissue engineering. By combining a mechanically stable, osteoinductive 3D-printed framework with sustained VEGF delivery to promote vascularization, this platform shows promise for treating critical-sized craniofacial and orthopedic bone defects.

**Supplementary Information:**

The online version contains supplementary material available at 10.1186/s12967-026-08090-5.

## Introduction

Critical-sized bone defects, whether arising from trauma, tumor resection, infection, or congenital malformations, remain among the most intractable challenges in orthopedic and craniofacial surgery [1–3]. Effective repair requires more than simply filling a void. It demands a coordinated orchestration of osteoconductive and osteoinductive signals together with a rapid and durable vascular response to deliver oxygen, nutrients, and progenitor cells [4–7]. Traditional solutions such as autografts and allografts have long been regarded as the clinical gold standard, but their effectiveness is constrained by donor site morbidity, limited availability, immunological concerns, and poor integration in large defects [8–13]. These shortcomings have fueled the pursuit of engineered biomaterial scaffolds that can replicate and enhance the body’s own regenerative processes.

Polymeric scaffolds, including poly(ε-caprolactone) (PCL), polylactic acid (PLA), and poly(lactic-co-glycolic acid) (PLGA), offer tunable degradation and mechanical properties but are biologically inert, limiting their capacity to drive bone formation on their own [14–17]. Bioactive ceramics such as hydroxyapatite (HA) bring excellent osteoconductivity but suffer from brittleness and difficulties in processing complex architectures [18–21]. Laponite is a synthetic clay, specifically a nanoscale material composed of sodium-magnesium silicate. Previous studies have demonstrated its ability to promote osteogenic differentiation, cell recruitment, and angiogenesis in bone defect models [22, 23]. Additionally, an appropriate amount of Laponite can activate the MAPK–ERK pathway, leading to improved bone repair outcomes [24]. However, the osteogenic ability of single-component Laponite is limited. Hydrogels, particularly gelatin methacryloyl (GelMA), excel at delivering bioactive signals and mimicking the extracellular matrix environment but lack the strength required in mechanically demanding sites [25–30]. Additionally, the encapsulated growth factor delivery is often dominated by early burst release, compromising long-term angiogenesis or tissue regeneration [31–34]. The challenge is therefore not simply one of selecting the “best” material, but of designing systems that bring together structural robustness, osteoinductive cues, and vascular support in an integrated and controllable way.

**Fig. 1:**
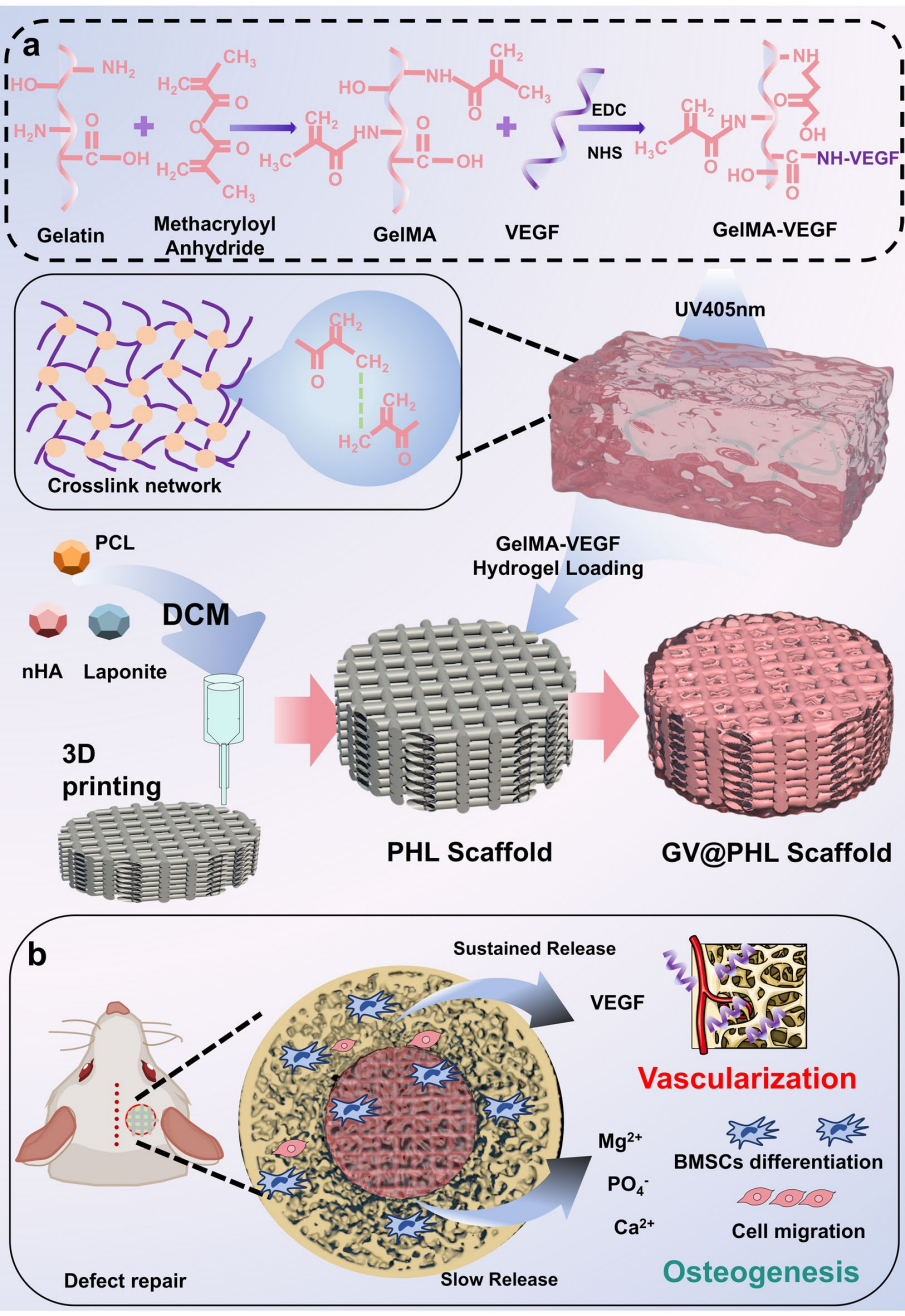
Schematic illustration of the preparation and application of GV@PHL composite scaffolds.** (a)** Vascular endothelial growth factor (VEGF) was covalently grafted onto gelatin methacryloyl (GelMA) to generate GelMA-VEGF hydrogel, which was crosslinked under ultraviolet (UV) irradiation at 405 nm. The PHL scaffold was fabricated via 3D printing of an equimolar mixture of polycaprolactone (PCL), nano-hydroxyapatite (nHA) particles, and Laponite dispersed in dichloromethane. Subsequently, the GelMA-VEGF hydrogel was loaded into the PHL scaffold, yielding the GV@PHL scaffold capable of simultaneously promoting osteogenesis and angiogenesis. **(b)** The GV@PHL scaffold was implanted into bone defects to facilitate defect regeneration

Three-dimensional (3D) printing has transformed this landscape by enabling precise fabrication of scaffolds with reproducible architectures, interconnected porosity, and customizable geometries. There are existing reports indicating that 3D-printed PCL scaffolds, by providing interconnected pores, support the infiltration of blood vessels into the scaffold and the formation of a network. The material is able to maintain long-term structural support, which is crucial for stable bone regeneration and the formation of a vascular network [35]. Additionally, it has been emphasized that when PCL scaffolds are combined with other bioactive materials, they provide excellent biocompatibility [36]. The technology opens doors for “patient-specific” 3D architecture designs and controlled spatial distribution of materials. In this work (Fig. 1), we report a two-component scaffold system capable of integrating a “vascularized-osteogenic microenvironment” within a 3D-printable, mechanically robust framework, coupled a covalently tethered, photo-crosslinkable angiogenic hydrogel. The first component, a PCL-nano-HA-Laponite (PHL) scaffold, was engineered to exhibit intrinsic osteogenic potential, while maintaining mechanical stability and the architectural fidelity necessary for cell infiltration and nutrient exchange. To complement this, an angiogenic hydrogel was introduced by covalently grafting vascular endothelial growth factor (VEGF) onto GelMA, followed by photo-crosslinking within the pores of the PHL scaffold (GV@PHL).

This approach provides sustained release of VEGF while preserving structural integrity, enabling simultaneous support of bone formation and vascularization. By integrating material mechanics, biological signaling, and fabrication precision, the GV@PHL platform exemplifies how composite scaffold systems can be designed to effectively repair tissue and promote both bone regeneration and vascularization.

## Materials and methods

### Material

The following materials were used in the fabrication of the GV@PHL scaffold: Polycaprolactone (PCL, Mw = 80,000), gelatin (type A, from cold fish skin), methacryloyl anhydride (MA), and Tris-HCl, all purchased from Sigma-Aldrich, while hydroxyapatite (HA), Laponite, and dichloromethane (99.5%, DCM) were obtained from Macklin. DMEM medium, osteogenic differentiation medium, the Cell Counting Kit-8, and the Live/Dead Cell Kit were purchased from Solarbio. The ALP staining kit, HUVECs, and mBMSCs were purchased from Gineo Biotechnology.

### Preparation of PHL ink

The PCL-nHA (PHA) ink was synthesized by the following protocol. HA particles and PCL pellets, with a mass ratio of 1:1, were first dissolved in a mixture of DCM and DBP at a ratio of 9:1. The mixture was then periodically stirred in a sealed container overnight until a homogeneous solution was achieved. Subsequently, Laponite powder was added to the mixture and stirred until the ink reached a shear stress viscosity of 30–40 Pa∙s, which is considered optimal for extrusion in 3D printing. For clarity and consistency in nomenclature, the synthetic PCL-nHA-Laponite ink used for 3D printing is referred to as PHL ink.

### Fabrication process of PHL scaffolds

In this work, a 3D printer (LN445, Guangzhou Maipu Regenerative Medicine Technology Co., LTD) was used to print PCL-nHA-Laponite (PHL) ink. To print the scaffold, biomaterial ink, composed of a specific concentration of Laponite, was dispensed by the extrusion system to form hydrogel filaments. The 3D structures were then fabricated by the moving system.

### Mechanical testing

The mechanical performance of the scaffolds (cylindrical in shape with a diameter of 1 cm and a height of 1 cm) was assessed using a universal testing machine (Shimadzu, EZ Test, Japan). Stress-strain curves were generated, and compressive strength and elastic modulus were calculated accordingly.

### Rheological characterization

Rheological properties of the biomaterial ink were measured using a rheometer (DISCOVERY HR-2) equipped with a cone-plate fixture (diameter: 40 mm, truncated gap distance: 50 μm, cone angle: 1.985°). Steady shear rate sweep tests were conducted on the inks within a shear rate range of 10⁻¹ to 10² s⁻¹ to characterize viscosity. For measuring the storage modulus (G′) and loss modulus (G″), frequency sweep tests were performed in the linear viscoelastic region at a strain of 1.0%. All tests were conducted at 37 °C to simulate physiological conditions.

### SEM analysis

Microstructures and morphologies of the PHL scaffold were observed using scanning electron microscopy (SEM, SU 8220, Hitachi, Tokyo, Japan) equipped with energy dispersive spectroscopy (EDS). Specifically, all specimens were sputter-coated with a 10 nm-thick gold layer under vacuum and imaged at an acceleration voltage of 5 kV. Energy spectrum scanning was simultaneously performed on the PHL scaffold to analyze its elemental composition.

### Fourier transform infrared spectroscopy detection

The FTIR (ALPHA, Bruker, Germany) spectra of samples were recorded in the wavenumber range of 4000 –400 cm^− 1^ with 32 scans per sample and a resolution of 4 cm^− 1^.

### Cell viability and proliferation assay

Cell viability was evaluated using the Cell Counting Kit-8 (CCK-8). To assess the cytotoxicity of the scaffold materials, mBMSCs were seeded in a 24-well plate with DMEM medium supplemented with 10% (v/v) FBS and 1% (v/v) penicillin-streptomycin, then incubated at 37 °C in 5% CO₂ overnight. The cells were subsequently incubated with the 48-h leaching solution of the scaffold materials for 1, 3, and 5 days, with the culture medium changed every 2 days. After 1, 3, and 5 days, the medium was removed, and CCK-8 solution was added to each well. Following incubation at 37 °C for 2 h, the absorbance was measured at 450 nm using a Thermo Fisher microplate reader.

At day 5, a Live/Dead Cell Kit was used to determine cell viability. mBMSCs at passage 5 (P5) were used in all experiments, and 2 × 10^4^ cells were seeded in each well of a 24-well plate. The cells were thoroughly washed with PBS and co-cultured with Live/Dead cell dye solution at 37 °C for 30 min, then observed using a fluorescence microscope (Zeiss Axiover200; Carl Zeiss).

### Cell migration

Cell migration was assessed using a wound healing assay. mBMSCs at passage 5 (P5) were used in all experiments, and 2 × 10^4^ cells were seeded in each well of a 24-well plate. A series of 1.5 mm linear scratches were created using a mold, which was removed after 12 h. The mBMSCs were then cultured with the leaching solution of the scaffold materials for 24 h. Wound closure was visualized under an optical microscope and analyzed using ImageJ software. The migration rate was calculated as the percentage of wound healing, defined by the formula:

$$\:Migration\:Rate=\frac{{S}_{1}-{S}_{0}}{{S}_{0}}\times\:100\%$$

where *S*_0_ is the initial scratch area and *S*_1_ is the final scratch area.

### Preparation of composite hydrogel scaffold

Briefly, gelatin (Sigma-Aldrich) from cold water fish was dissolved in PBS at 50 °C and stirred to prepare a 10% (w/v) homogeneous solution. Methacrylic anhydride (MA, Sigma-Aldrich; 0.1 mL per gram of gelatin) was added to the homogeneous gelatin solution at a rate of 0.5 mL/min with continuous stirring. The mixed solution was allowed to react at 50 °C for 3 h with stirring [37, 38]. The resulting GelMA solution was then poured into 8–14 kDa cutoff dialysis tubing (VWR Scientific USA) and dialyzed against deionized water at 50 °C for 6 days to remove unreacted MA and byproducts, with the deionized water replaced every 1–2 days. The resulting GelMA solution was frozen overnight at − 80 °C, lyophilized, and stored at − 20 °C for further use.

For GelMA-COOH synthesis, the mixture was stirred overnight, diluted with 50 mL PBS, and dialyzed against deionized water using 3.5 kDa cutoff dialysis tubing for 10 days at room temperature to remove impurities. The solution was lyophilized to yield GelMA-COOH as a white porous foam. For VEGF conjugation, 300 mg of GelMA-COOH was dissolved in 5 mL PBS, followed by the addition of 0.5 mg EDC and 0.5 mg NHS, which were agitated for 30 min before VEGF addition. The mixture was agitated at room temperature for 6 h, then dialyzed against deionized water using 3.5 kDa cutoff dialysis tubing and lyophilized to generate GelMA-VEGF. Prepare a photo-initiator (LAP) solution at 0.25% (w/v). Add GelMA to obtain a final concentration of 15% (w/v). Photocrosslink the solution under 405 nm blue light irradiation at room temperature (10 mW/cm²) for 15 s to obtain the GelMA-VEGF hydrogel.

### Alizarin red staining (ARS)

mBMSCs at passage 5 (P5) were used in all experiments, and 2 × 10^4^ cells were seeded in each well of a 24-well plate containing different materials and induced with osteogenic medium for 7, 14, and 21 days. ARS staining reagent was added, and the samples were incubated at room temperature for 20 min. Excess ARS was then washed away with distilled water, and images of the stained cells were captured using an optical microscope.

### Alkaline phosphatase (ALP) staining

mBMSCs at passage 5 (P5) were used in all experiments, and 2 × 10^4^ cells were seeded in each well of a 24-well plate containing different materials and induced with osteogenic medium for 7, 14, and 21 days. ALP staining reagent was added, and the samples were incubated at room temperature for 30 min. Excess ALP was then washed away with distilled water, and images of the stained cells were captured using an optical microscope.

### Animals and surgical procedures

In the animal experiments, three animals were included in each experimental group (*n* = 3), and each animal was considered an independent biological replicate.

Animals were allocated to experimental groups using a simple randomization method. To minimize potential bias, all outcome assessments and data analyses were performed by investigators who were blinded to the group assignments throughout the study.

Male SD rats (250 ± 20 g, 8–10 weeks old) were used in this work. All surgical procedures were performed under 4% isoflurane anesthesia. After hair removal using an electric clipper, surgical sites were sterilized with povidone-iodine solution. A critical-sized calvarial defect (5 mm diameter) was created along the midline of the calvarium using a dental trephine bur to establish the calvarial defect model.

The rats were randomly divided into three groups: (1) Defect control group (Control), (2) PHL Scaffold group, and (3) GV@PHL Scaffold group (PHL Scaffold + GelMA-VEGF). At 8 weeks after surgery, blood samples were collected from the main abdominal vein, and vital organs along with regenerated tissue from the defect area were harvested for further evaluation.

### Micro CT analysis

Micro-CT was used to quantify new bone formation in a 5 mm diameter circular calvarial defect.

At 6 weeks after surgery, SD rats were euthanized via an overdose of pentobarbital anesthesia. The intact skulls were removed and stored in 4% (w/v) paraformaldehyde (PFA) solution for further analysis. The skulls were analyzed using micro-CT, and a 5 mm diameter ring on the implant surface was defined as the volume of interest (VOI). Bone volume fraction (BV/TV), bone mineral density (BMD), bone surface area density (BS/TV), and trabecular number (Tb.N) within the VOI were quantified.

Acquisition parameters. Specimens were scanned at 65 kV and 76 µA with an isotropic voxel size of 36 μm. A 0.5-mm Al filter was applied to reduce beam-hardening artifacts. Scans were acquired over 360° rotation with a 0.5° rotation step, 300 ms exposure time, and frame averaging = 2. Reconstruction. Projection data were reconstructed using the vendor’s reconstruction software. Beam-hardening correction was set to 30%, and ring-artifact reduction was set to 10 to improve image quality.

BMD calibration. For bone mineral density (BMD) quantification, datasets were calibrated using hydroxyapatite (CaHA) phantoms with known densities of 0.25 and 0.75 g·cm⁻³, scanned using the same settings as the specimens. Calibration was performed in the analysis software to convert grayscale values to mgHA·cm⁻³.

ROI definition. A cylindrical region of interest (ROI) was defined by a circle of 5.0 mm diameter centered on the original defect and spanning a fixed height of 0.90 mm (corresponding to 25 slices at 36 μm). The ROI was positioned to match the surgical defect boundaries while avoiding surrounding native cortical bone as much as possible.

Segmentation and outcome measures. Mineralized bone within the ROI was segmented using a fixed global threshold (22,000) applied consistently to all samples. Three-dimensional morphometric parameters were then computed, including tissue volume (TV), bone volume (BV), bone volume fraction (BV/TV), and mean BMD within the ROI.

### Histological analysis

Harvested skull specimens were fixed and decalcified in 10% (w/v) ethylenediamine tetra-acetic acid (EDTA) for two months, then dehydrated through a graded alcohol series and embedded in paraffin. The central segment was sectioned into 10-µm-thick slices using a rotary microtome (Leica, Hamburg, Germany). Hematoxylin and eosin (H&E) staining and Masson’s Trichrome staining were performed on paraffin sections according to standard protocols, and images were acquired using bright-field microscopy. For organ specimens, H&E staining was similarly performed on paraffin sections and observed under bright-field microscopy.

### Blood analysis

Blood samples were placed into designated collection tubes for complete blood count (CBC) and biochemical analysis. At 6 weeks after surgery, animals were euthanized, and serum was harvested for analysis. Serum levels of alanine aminotransferase (ALT), aspartate aminotransferase (AST), and creatinine (CR) were measured using a Siemens ADVIA 2400 biochemistry analyzer. Counts of whole blood cells, including white blood cells (WBC), red blood cells (RBC), platelets (PLT), and hematocrit (HCT), were determined using a SYSMEX whole blood analyzer (Japan).

### Statistical analysis

Values are presented as mean ± standard deviation (SD) unless otherwise specified in the figure legends. Statistical significance between two groups was analyzed using two-tailed Student’s t-tests. For multiple comparisons, one-way analysis of variance (ANOVA) with Tukey’s post hoc test was employed. Statistical analyses were performed using GraphPad Prism software. A *p*-value < 0.05 was considered significant, with notation: *p < 0.05, **p < 0.01, ***p < 0.001, ****p < 0.0001.

## Results and discussion

### Printability analysis of ink

**Fig. 2:**
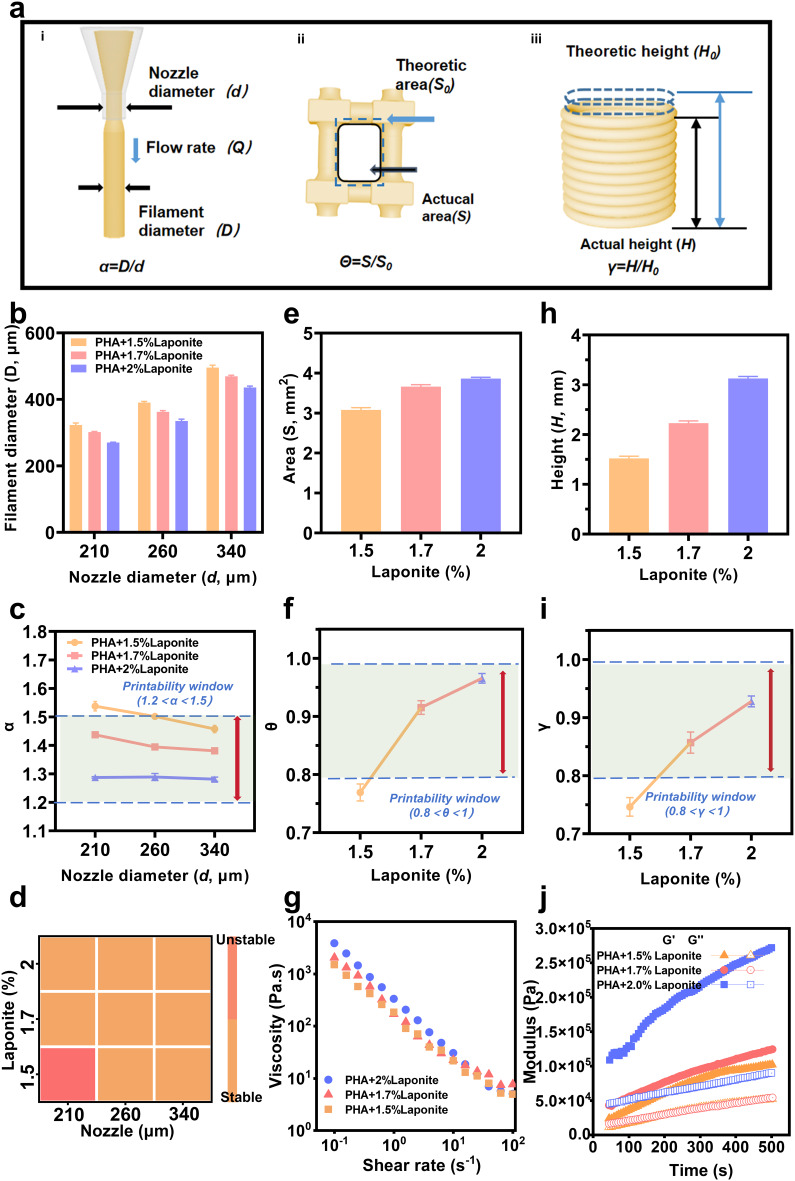
Analysis of 3D printing ink fidelity. **a**) Printability analysis of 3D printing ink during extrusion Design mode. i: Schematic diagram and definition of printing ink expansion phenomenon: α = D/d. ii: Schematic diagram and definition of diffusion phenomenon in printed grid structures: θ = S/S₀. iii: Schematic diagram and definition of sinking phenomenon in the height direction: γ = H/H₀. **b-c**) Effect of the nozzle diameter on (figure **b**) the extruded filament diameter, and (figure **c**) α. **d**) Heatmap of the relationship between nozzle diameter, ink concentration, and 3D printing stability. **e**) The actual area of the printed grid printed with different concentrations of ink. **f**) Printable window for printing the grid structure with different concentrations of ink. **h**) Actual height of the cylindrical model with different concentrations of ink. **i**) The table window for different ink printing cylinder models. **g**) Viscosity and shear rate of the ink diagram. **j**) Energy storage modulus and loss modulus of the ink The sample size for all experiments is *n* = 3

To fabricate scaffolds with good shape fidelity, it is essential to evaluate the printability of the PHL ink to determine the optimal printing parameters [39]. During the extrusion process, the ink expanded upon extrusion from the nozzle due to the release of shear stress, resulting in a filament diameter larger than that of the nozzle (Fig. 2a i). Here, the α value describes the degree of filament expansion (α = *D*/*d*, where *D* is the filament diameter, and *d* is the nozzle inner-diameter). The degree of expansion was influenced by both ink concentration and nozzle diameter, and these results are shown in Fig. 2b. Figure 2b demonstrates that the diameter of the extruded filament for PCL-nHA-2% Laponite is closest to the inner diameter of the nozzle, irrespective of the nozzle diameter.

Figure 2c reveals that the *α* value decreases with increasing nozzle diameter; furthermore, inks containing higher concentrations of nanoclay exhibited less expansion. This is attributed to the fact that ink passing through a smaller-diameter nozzle experiences greater shear stress at a higher flow rate, thereby resulting in more strain. Consequently, higher concentrations of nanoclay require a greater yield stress for deformation. Here, we defined ‘1.2 < α < 1.5’ as the printable range, and the printability window for the extrusion process was determined as presented in Fig. 2c. Meanwhile, heatmap results indicate that structural instability during printing only occurs when PCL-nHA-1.5% Laponite ink is extruded using a nozzle with a 210 μm inner diameter (Fig. 2d).

During the stacking process, intersecting filaments diffuse during grid structure printing due to ink accumulation (Fig. 2a ii), and the scaffold undergoes vertical sagging under the influence of gravity (Fig. 2a iii). Here, the *θ* value describes the degree of diffusion (*θ = S/S₀*, where *S* represents the actual area and *S₀* denotes the theoretical area), while the *γ* value describes the degree of sagging (*γ = H/H₀*, where *H* is the actual height and *H₀* is the theoretical height). An effective strategy to reduce both diffusion and sagging is to increase the nanoclay concentration, which directly enhances the material’s resistance to deformation. These results are presented in Fig. 2e, h. Here, we defined ‘0.8 < θ < 1.0’ and ‘0.8 < γ < 1.0’ as the printable range, and the printability window for the stacking process was determined as presented in Fig. 2f, i. To verify whether the biomaterial ink meets the requirements of extrusion-based 3D printing, specifically, shear-thinning and rapid gelling behaviors, the rheological properties of PHL inks with varying Laponite concentrations were characterized. PHL inks with different concentrations exhibit shear-thinning behavior (Fig. 2g). 

Subsequently, modulus tests were conducted, revealing that the storage modulus (G′) is greater than the loss modulus (G″): the bulk phase exhibits elastic solid characteristics, i.e., it forms a gel-like structure. This is favorable for ensuring that the printed scaffold maintains excellent shape fidelity and stabilizes the model’s basic shape during the printing process (Fig. 2j).

### Shape fidelity and performance verification of printing inks

**Fig. 3:**
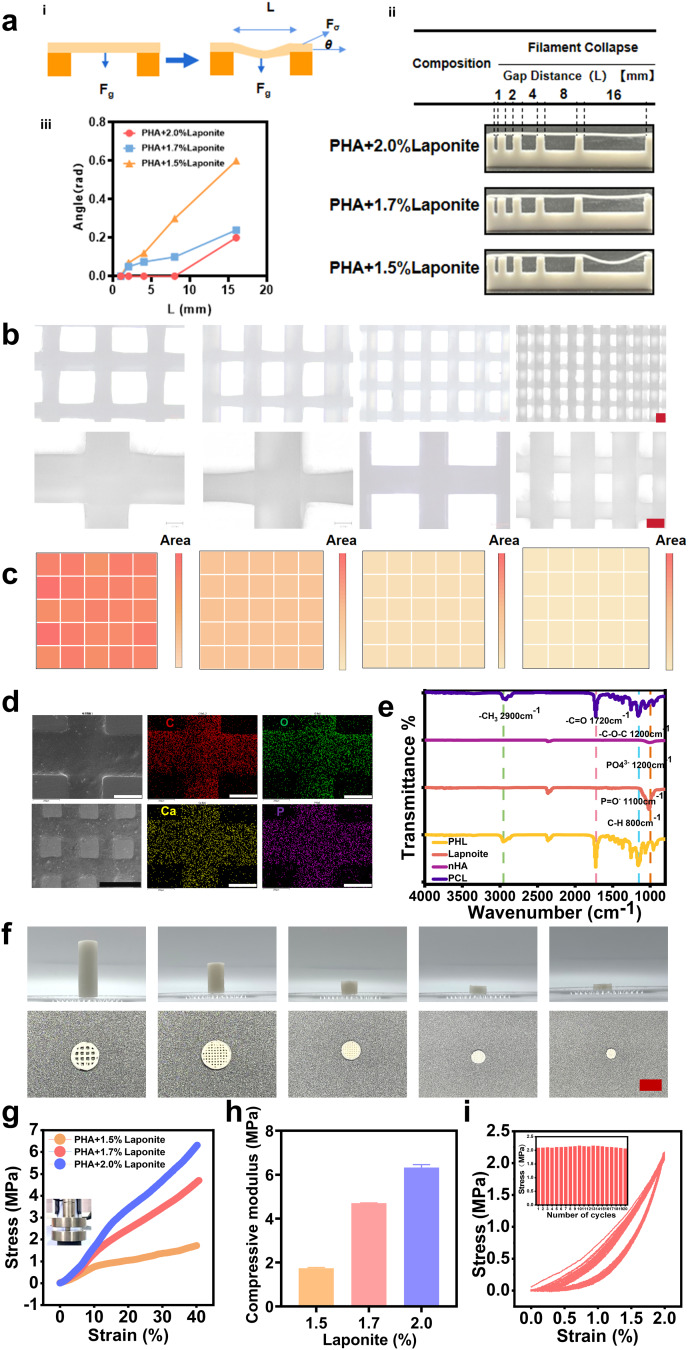
3D printing ink performance.** (a)** Print line collapse experiment. i: Print line collapse experiment diagram. ii: Photo shows the fiber appearance of PHA ink lines with different Lap concentrations on the support beam iii: Quantitative statistical chart of angles. **(b)** Optical micrograph of the grid bracket with different porosity (scale bar=200 μm). **(c)** Print a heat map of both grid areas. **(d)** The two images on the left are SEM images and the four images on the right are energy spectrum scans (C, O, Ca, P) (black scale bar = 1 mm, white scale bar = 250 μm. **e)** Infrared spectrum of 3D printing scaffold materia. **f)** Printing photos of cylindrical supports with different heights and diameters (scale bar=1 cm). **g)** Stress and strain curve of printed Scaffold with different concentrations of ink. **h)** Compression modulus of printed Scaffold with different concentrations. **i)** Anti-fatigue test of Scaffold The sample size for all experiments is *n* = 3

The filament collapse test (Fig. 3a i) revealed that filament sag increases with gap length, as indicated by the increase in deflection angle *θ*. Moreover, sag decreased with increasing nanoclay concentration and yield stress (Fig. 3a ii and iii). The resulting linear regression plots for different concentrations revealed that the slope of each line decreased with increasing nanoclay concentration (and thus increasing yield stress; Fig. 3a iii). The results demonstrated that the mechanical properties of PHL (PCL-nHA-Laponite) were enhanced with increasing Laponite concentration, indicating its potential for printing hollow structures. The deflection angle of PCL-nHA-2% Laponite is the smallest, indicating that the material exhibits excellent structural strength and printability.

Based on computer-aided design (CAD) of the structure, a representative cylindrical. stl structure with adjustable pore parameters was constructed and pre-imported into a 3D printing system (Model: LN445, Maipu). The PCL-nHA-2% Laponite ink was loaded into a printing nozzle for printing at different scales. During printing, parameters including extrusion pressure (150–350 kPa), nozzle inner diameter (260 μm), vertical distance between the nozzle tip and printing platform (0.3–0.4 mm), and printing density were controlled to fabricate 3D mesh structures. After printing, the microscopic surface morphology of the mesh scaffold was characterized using an optical microscope (Fig. 3b). The vascularization capacity of scaffold materials might be closely related to their pore characteristics. A reasonable pore structure, favorable mechanical properties, stable degradation rate, and effective biological inductivity are conducive to cell adhesion, migration, and neovascularization. Therefore, we printed scaffolds with different porosities and quantified the uniformity of their grid areas. Heatmap-based quantification of grid area uniformity for printed scaffolds with different porosities showed that the grid areas fabricated using PHL ink were uniform and structurally stable (Fig. 3c). Meanwhile, the surface elemental composition of osteoconductive scaffolds must be evaluated with respect to factors including bone tissue compatibility, biological activity, and scaffold performance. Therefore, we performed energy-dispersive spectroscopy (EDS) analysis on the printed scaffolds to confirm two findings: (1) the presence of osteogenically essential elements (C, O, Ca, P) and (2) the uniform distribution of these elements across the scaffold surface. These characteristics ensure the scaffold surface exhibits excellent biocompatibility, osteoconductive potential, and mechanical properties, thereby better facilitating bone tissue growth and healing (Fig. 3d).

FTIR spectroscopy was used to analyze polycaprolactone (PCL), hydroxyapatite (HA), and Laponite RD (Laponite). For PCL: the carbonyl (C = O) absorption peak is observed at ~ 1720 cm⁻¹, and methylene (-CH₂-) and methyl (-CH₃) stretching vibrations occur at 2940 –2867 cm⁻¹. The C-O-C stretching vibration of PCL is observed at 1160–1240 cm⁻¹. For HA: phosphate (PO₄³⁻) absorption peaks are observed at ~ 1200 –900 cm⁻¹, and phosphate-hydroxyl (PO₄³⁻-OH⁻) bonding vibrations occur at 1000 –600 cm⁻¹. For Laponite: stretching vibrations of the P = O bond appears at 1200 –1000 cm⁻¹, and aromatic ring C-H bending vibrations occur at 1000 –700 cm⁻¹. When PCL, HA, and Laponite are fabricated to form the PHL material, superposition of the three components’ characteristic peaks is observed (Fig. 3e).

A universal testing machine was used to characterize the stress-strain behavior of three groups of PCL-nHA scaffolds with varying Laponite concentrations. The PCL-nHA-2% Laponite scaffold reached a stress of 6 MPa at 40% strain (Fig. 3g, h). To further evaluate the scaffold’s fatigue resistance, the PCL-nHA-2% Laponite scaffold was compressed to 2% strain at a rate of 2 mm·s⁻¹, followed by load release at the same rate. The scaffold was subjected to 20 consecutive cyclic loading cycles, and it exhibited excellent fatigue resistance without significant deformation (Fig. 3i). The prepared PCL-nHA-2% Laponite ink was used to 3D-print cylindrical scaffolds of varying heights and diameters. The printed scaffolds exhibited complete structures and high shape fidelity (Fig. 3f).

### Biocompatibility performance and in vitro osteogenic effects of PHL scaffold

**Fig. 4:**
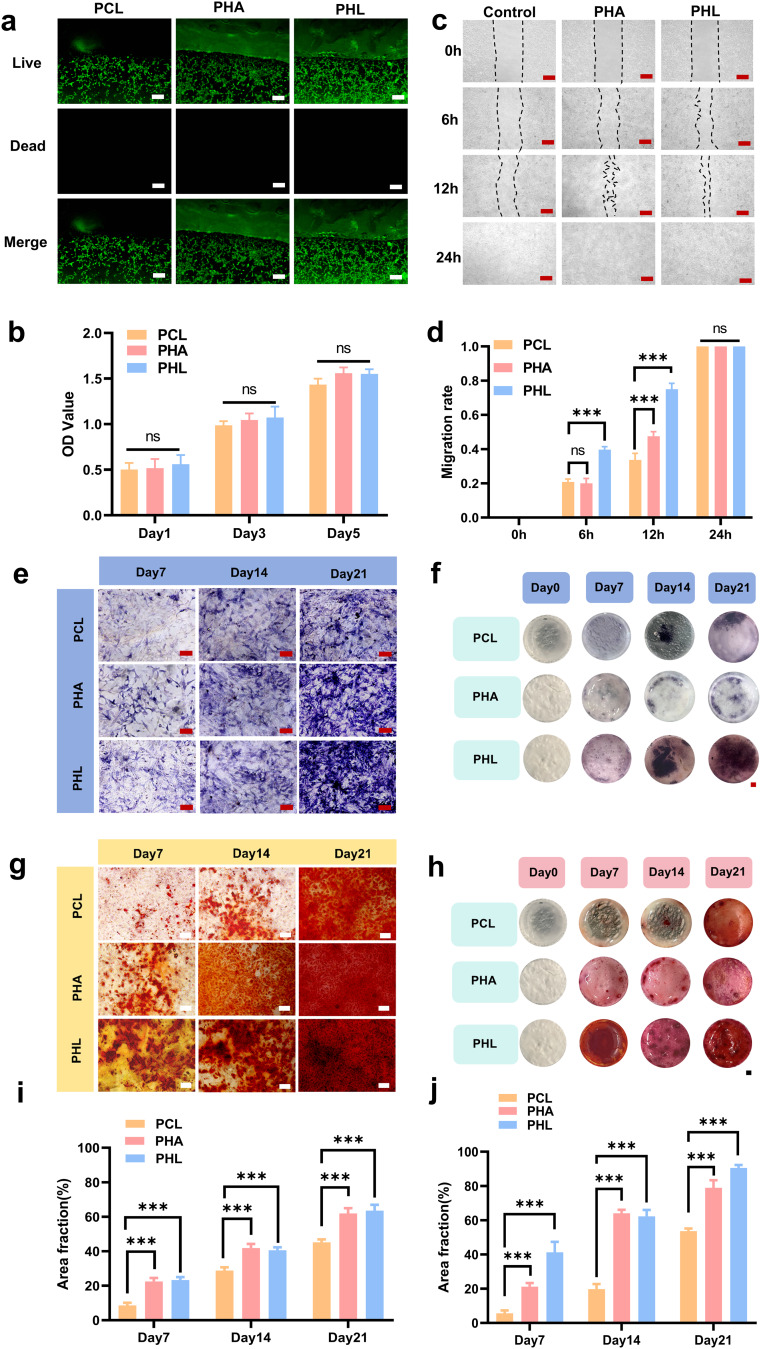
Characterization of biocompatibility and osteogenic inductive capacity of scaffold materials.** (a)** Cytotoxicity assessment of PCL, PHA, and PHL scaffolds against bone marrow mesenchymal stem cells (BMSCs) via Live/Dead staining. Green: Calcein-AM (live cells), Red: Propidium iodide (PI, apoptotic cells) (Scale bar = 200 μm). **(b)** Proliferation and viability of BMSCs co-cultured with scaffolds for 1, 3, and 5 days, determined by CCK-8 assay. **(c)** Representative images of scratch wound healing assay (Scale bar = 200 μm). **(d)** Quantitative analysis of cell migration rate. **(e)** Alkaline phosphatase (ALP) staining of BMSCs after osteogenic differentiation induction (Scale bar = 200 μm). **(f)** ALP staining of BMSCs directly co-cultured with scaffolds without osteogenic supplements(Scale bar=1 mm). **(g)** Alizarin red S (ARS) staining of BMSCs after osteogenic differentiation induction (Scale bar = 200 μm). **(h)** ARS staining of BMSCs directly co-cultured with scaffolds without osteogenic supplements(Scale bar=1 mm). **(i)** Quantitative analysis of ALP-positive staining area. **(j)** Quantitative analysis of ARS-positive mineralized nodule area. All experimental data are expressed as mean ± SD (*n* = 3). *p < 0.05, **p < 0.01, ***p < 0.001

Bone marrow mesenchyml stem cells were co-cultured with scaffolds, and cell viability and proliferation were evaluated using live/dead cell staining and the CCK-8 (Cell Counting Kit-8) assay to assess the scaffolds’ biocompatibility. On day 1, the cells exhibited significant proliferation. The live/dead cell staining showed high numbers of viable cells in all groups (Fig. 4a). The CCK-8 assay results showed a gradual increase in absorbance with extended incubation periods, indicating that mBMSCs (mouse bone marrow mesenchymal stem cells) were proliferating (Fig. 4b). After co-culturing with mBMSCs for 24 h, PCL, PHA (PCL-nHA), and PHL(PCL-nHA-Laponite) materials induced no cell death. Adding specific concentrations of nHA (nano-hydroxyapatite) and Laponite to PCL did not impair the normal proliferation of mBMSCs.

mBMSCs were cultured in scaffold extracts, and we performed a scratch wound healing assay to evaluate mBMSC migration, a commonly used method that the process closely associated with in vitro osteogenic repair, and assess the scaffolds’ pro-osteogenic potential. As shown in Fig. 4c, at 6 and 12 h post-scratching, mBMSC migration was significantly promoted by PHL (PCL-nHA-Laponite). Notably, mBMSCs exhibited a migration rate of 77% ± 2% after 12 h of treatment with PHL (PCL-nHA-Laponite). In contrast, the migration rate in the control group was only ~ 20% (*p* < 0.001) (Fig. 4d).

To evaluate the osteogenic potential of BMSCs expanded in vitro using the scaffold culture system, we switched the growth medium to osteogenic differentiation medium after 7 days of expansion. We found that osteogenic differentiation was significantly upregulated in the PHL (PCL-nHA-Laponite) culture system, as evidenced by both alkaline phosphatase (ALP) staining (Fig. 4e, f) and alizarin red S (ARS) staining (Fig. 4g, h), two established markers of osteogenesis. We quantified the ALP and ARS staining areas of cells within the field of view, and the results showed that the staining area in the PHL (PCL-nHA-Laponite) group was greater than that in the other two groups (Fig. 4i, j). After 21 days of induction, ALP expression exhibited a similar trend, with the enzyme activity in the PHL group being four times that of the PCL group and twice that of the PHA group (Fig. S1).

The addition of Laponite to the ink not only enhances the scaffold’s structural integrity and shape fidelity, but also promotes the osteogenic differentiation of mBMSCs. This is attributed to Laponite’s ability to induce osteogenic differentiation of bone marrow mesenchymal stem cells (BMSCs) and human adipose-derived stem cells (hADSCs), even in the absence of exogenous protein factors. However, Laponite’s powdery structure cannot be directly used for bone tissue engineering applications. Therefore, the PHL scaffold not only enhances the scaffold’s osteogenic inductivity but also imparts mechanical properties to the scaffold.

### Endowing PHL scaffolds with vascularization capacity

Vascularization is a critical prerequisite for effective osteogenesis, as sufficient blood supply delivers essential nutrients, oxygen, and osteogenic precursor cells to the defect site, addressing a major challenge in critical-sized bone defect repair [40–42]. In the present work, we developed PCL-nano-HA-Laponite (PHL) scaffolds that demonstrated intrinsic osteogenic potential and could be 3D-printed to form interconnected porous architectures, providing an ideal framework for subsequent biological functionalization. We herein designed a synergistic strategy to further augment their therapeutic efficacy. Specifically, we covalently grafted vascular endothelial growth factor (VEGF) onto gelatin methacryloyl (GelMA) via stable chemical bonds, constructing a photo-crosslinkable angiogenic GelMA-VEGF hydrogel. This hydrogel was then photo-embedded into the interconnected porous structure of PHL scaffolds, leveraging light stimulation for precise integration. The goal of this design was to create a favorable vascularized microenvironment within the scaffold, which would work in synergy with the PHL scaffold’s intrinsic osteogenic properties to significantly enhance overall bone regeneration capacity, representing a tailored solution to coordinate vascularization and osteogenesis for improved repair outcomes.

**Fig. 5:**
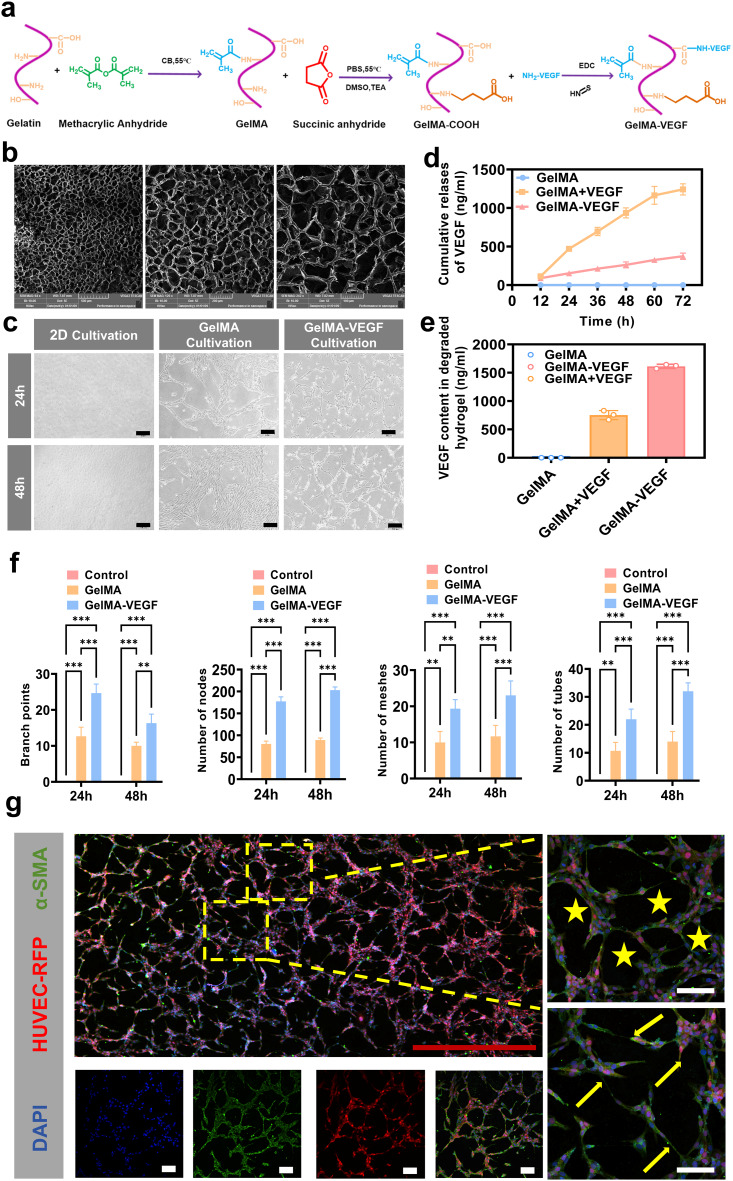
Synthesis, characterization and angiogenic evaluation of GelMA-VEGF hydrogel. (**a**) Chemical formula for the synthesis of GelMA-VEGF hydrogel. (**b**) Scanning electron microscopy (SEM) images of the hydrogel with scale bars of 500 μm, 200 μm, and 100 μm from left to right. (**c**) Digital images of HUVECs differentiating into vascular structures under different culture conditions (Scale bar = 200 μm). (**d**) Quantitative analysis of VEGF release over time. (**e**) Quantitative analysis of residual VEGF in the hydrogel. (**f**) Quantitative analysis of the degree of vascularization of HUVECs on the hydrogel. (**g**) Immunofluorescence images of the differentiation of HUVEC-RFP co-cultured with GelMA-VEGF hydrogel (red Scale bar = 1000 μm, white Scale bar = 100 μm). All experimental data are expressed as mean ± SD (n = 3). *p < 0.05, **p < 0.01, ***p < 0.001

VEGF was conjugated to GelMA via N’-ethylcarbodiimide hydrochloride (EDC)/N-hydroxysuccinimide (NHS) coupling chemistry (Fig. 5a). The amount of human VEGF conjugated to GelMA hydrogels could be quantified using a human VEGF ELISA kit. Furthermore, the release of VEGF from GelMA-VEGF hydrogels was compared with that from physically mixed GelMA/VEGF samples (GelMA+VEGF) and pure GelMA hydrogels. As anticipated, the amount of VEGF released from GelMA-VEGF hydrogels was approximately fourfold lower than that from physically mixed GelMA + VEGF samples at the first 3 days (Fig. 5d). The amount of VEGF remaining in the hydrogel matrix after 3 days was quantified by degrading the matrix with collagenase and measuring the VEGF released into the solution. During the slow degradation of the hydrogels, the amount of VEGF released from GelMA-VEGF hydrogels was sustainably higher than that from physically mixed GelMA+VEGF samples, thus confirming the advantage of chemical conjugation between VEGF and the hydrogel network (Fig. 5e). The VEGF dose is 2000 ng/mL [43]. During the entire release process, the GelMA+VEGF group released approximately 60% of the total dose over 72 h, while the GelMA-VEGF group released approximately 20% over 72 h (Fig. S2).

Subsequently, GelMA-VEGF hydrogels were imaged using scanning electron microscopy (SEM). The results showed that the hydrogels exhibited a porous structure similar to the nature extracellular matrix, which is conducive to mass transport (Fig. 5b) [44–46]. In this work, human umbilical vein endothelial cells (HUVECs) were used to evaluate the angiogenic potential of GelMA-VEGF hydrogel via tube formation assays. The synthesized pure GelMA and the 2D cultured HUVECs were set as the control group. As shown in Fig. 5c, HUVECs cultured on GelMA-VEGF hydrogels exhibited a significantly greater ability to form the significant capillary-like network features, surpassing that of the control group. Furthermore, a detailed analysis of quantitative metrics, including the number of meshes, branches, nodes, and tubes, revealed that the GelMA-VEGF group exhibited substantially higher values compared to the control group. This marked improvement in angiogenic parameters underscored the superior performance of GelMA-VEGF hydrogel constructs in facilitating vascular network formation (Fig. 5f).

After 24 h of culture, we performed detailed immunofluorescence imaging of HUVECs expressing red fluorescent protein (HUVEC-RFP) cultured on GelMA-VEGF hydrogels. We used an α-smooth muscle actin (α-SMA) antibody to stain smooth muscle actin, 4’,6-diamidino-2-phenylindole (DAPI) to stain cell nuclei, and combined these with the red fluorescent protein (RFP) labeling of HUVEC-RFP. This allowed clear observation of HUVEC-RFP sprouting and tube formation across the entire field of view (Fig. 5g). Luminal structures were marked with star symbols, and cell sprouting was marked with arrows. These results further confirmed that GelMA-VEGF hydrogels exhibit potential capacity to promote vascularization.

### In vivo osteogenic effects of the GV@PHL scaffold

**Fig. 6:**
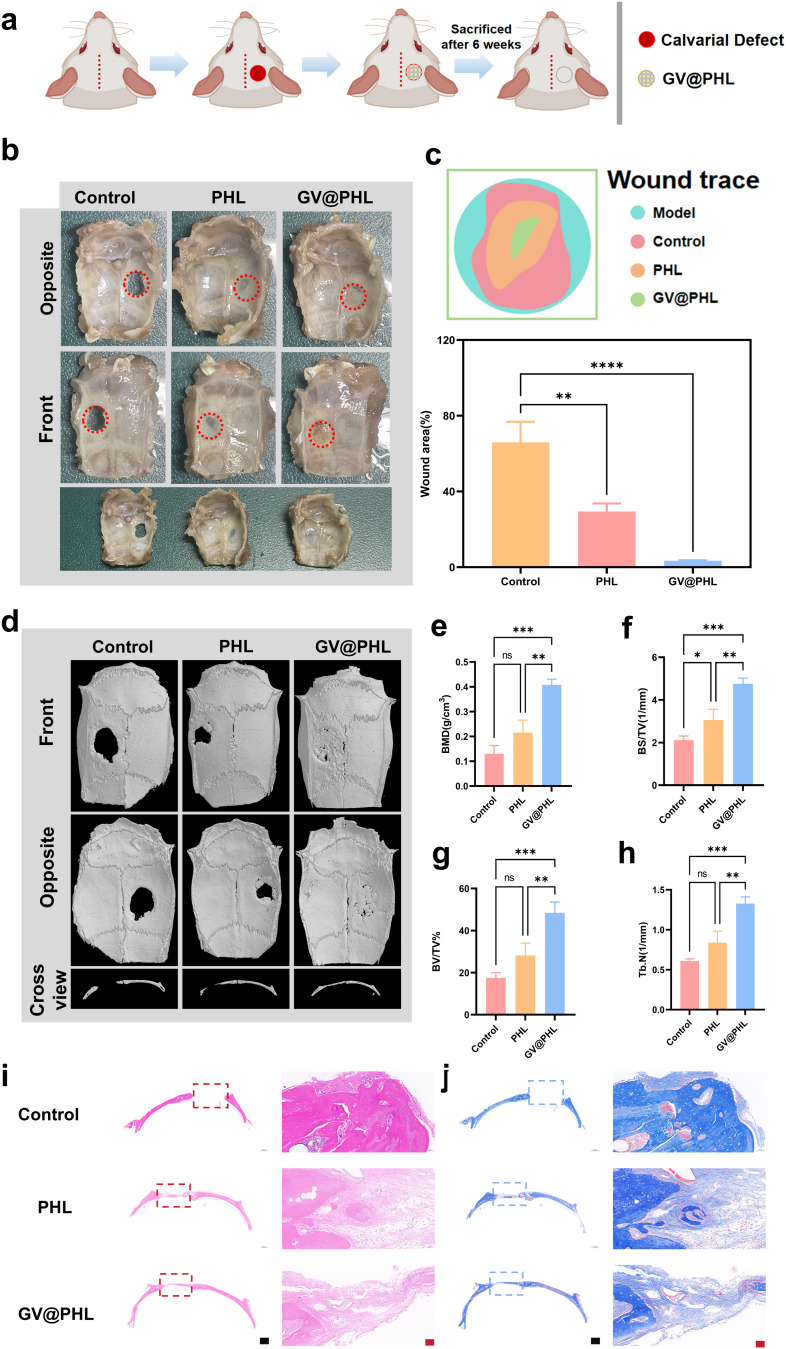
Osteogenic promotion of GV@PHL scaffold in vivo. (**a**) Schematic illustration of critical-size bone defect modeling in SD rats and GV@PHL transplantation. **(b)** Macroscopic images of rat calvarial bone defects treated with different scaffolds at 6 weeks. **(c)** Bone defect traces and defect rates of each experimental group after various treatments. **(d)** Micro-CT 3D reconstruction images of rat calvarial bone defects treated with different scaffolds at 6 weeks post-surgery. **e-h)** Bone mineral density (BMD), bone volume/tissue volume (BV/TV), bone surface/total volume (BS/TV), and trabecular number (Tb.N) of bone defects treated with different scaffolds. **i-j)** HE staining and Masson trichrome staining results of each group (black scale bar = 1 mm, red scale bar = 50 μm). All experimental data are expressed as mean ± SD (*n* = 3). *p < 0.05, **p < 0.01, ***p < 0.001

Building on the in vitro validation that PHL scaffolds possess osteogenic potential and GelMA-VEGF (GV) hydrogels enable sustained VEGF release to promote vascularization, we further integrated these two components into a synergistic composite system, GV@PHL, to address the critical challenge of coordinating vascularization and osteogenesis in bone defect repair. Unlike conventional scaffold designs that either focus on osteogenic support or vascularization alone, our strategy leverages 3D printing to construct PHL scaffolds with interconnected pores (facilitating cell infiltration and nutrient exchange) and photo-crosslinking to embed GV hydrogels into these pores. This deliberate integration ensures that the GV hydrogel’s pro-angiogenic capacity (via sustained VEGF release) works in tandem with the PHL scaffold’s structural stability and osteogenic inductivity, creating a “vascularized-osteogenic microenvironment” that addresses both nutrient supply and bone formation simultaneously. To verify the therapeutic efficacy of this innovative composite system, we conducted in vivo studies using rat calvarial defect models (Fig. 6a). 

After establishing the animal models and implanting the different scaffold groups, we assessed wound healing in SD rats at 6 weeks using macroscopic images (Fig. 6b). We found that the GV@PHL group exhibited a smaller wound area than the other groups. The healing map revealed that the GV@PHL group had almost completely healed, with less than 5% of the initial wound area remaining after 6 weeks; in contrast, the other groups showed distinct regions of incomplete healing (Fig. 6c). At 6 weeks post-surgery, rat skulls were harvested and analyzed using micro-computed tomography (micro-CT). As shown in Fig. 6d, the images revealed new bone formation around the defect edges, which had extended to the center of the bone defect cavity in the GV@PHL group. In contrast, only a small amount of new bone formed at the defect edges in the control group. In the PHL group, bone ingrowth was observed at the defect edges after 6 weeks of healing; however, the healing effect was less pronounced compared to the GV@PHL group. Quantitative micro-CT analysis was performed accordingly (Fig. 6d), quantifying bone mineral density (BMD), bone volume fraction (BV/TV), trabecular number (Tb.N), and bone surface density (BS/TV).

Analysis of the quantitative results showed that within the 5 mm-diameter circular defect area, the BMD of newly formed bone was lowest in the control group, higher in the PHL scaffold group, and highest in the GV@PHL composite scaffold group, with the latter exhibiting the highest BMD in the repaired bone (Fig. 6e). Analysis of relative bone volume (BV/TV) in the repair area also reflected this trend. BV/TV (Fig. 6f) directly indicates bone volume changes within the area, with the control group showing the lowest values, the PHL scaffold group showing higher values, and the GV@PHL composite scaffold group showing the highest values. Bone surface density (BS/TV) indirectly reflects bone volume within the area (Fig. 6g), while trabecular number (Tb.N) represents the average number of intersections between bony and non-bony tissue (Fig. 6h). These results confirmed that the GV@PHL composite scaffold exhibited the most robust repair efficacy.

Histological analysis was first performed at 6 weeks post-surgery to assess the degree of calvarial defect repair. As shown in Fig. 6i, hematoxylin and eosin (H&E) staining revealed a smaller residual defect size and more newly regenerated bone in the GV@PHL group compared to the blank control and PHL groups. Notably, in the GV@PHL group, newly formed bone almost completely bridged the defect, suggesting a robust bone regeneration capacity. In magnified H&E-stained images, more newly formed blood vessels were observed in the GV@PHL group, indicating that the GV@PHL scaffold also exhibits excellent capacity to promote neovascularization. The entire animal experiment process was supervised by the Experimental Animal Welfare and Ethics Committee of Kunming Medical University. The study passed the welfare and ethics review, and the ethical review approval number is KMMUD2025006. All procedures were conducted in accordance with the guidelines for the care and use of laboratory animals.

### In vivo safety evaluation of GV@PHL scaffolds following transplantation in rats

**Fig. 7:**
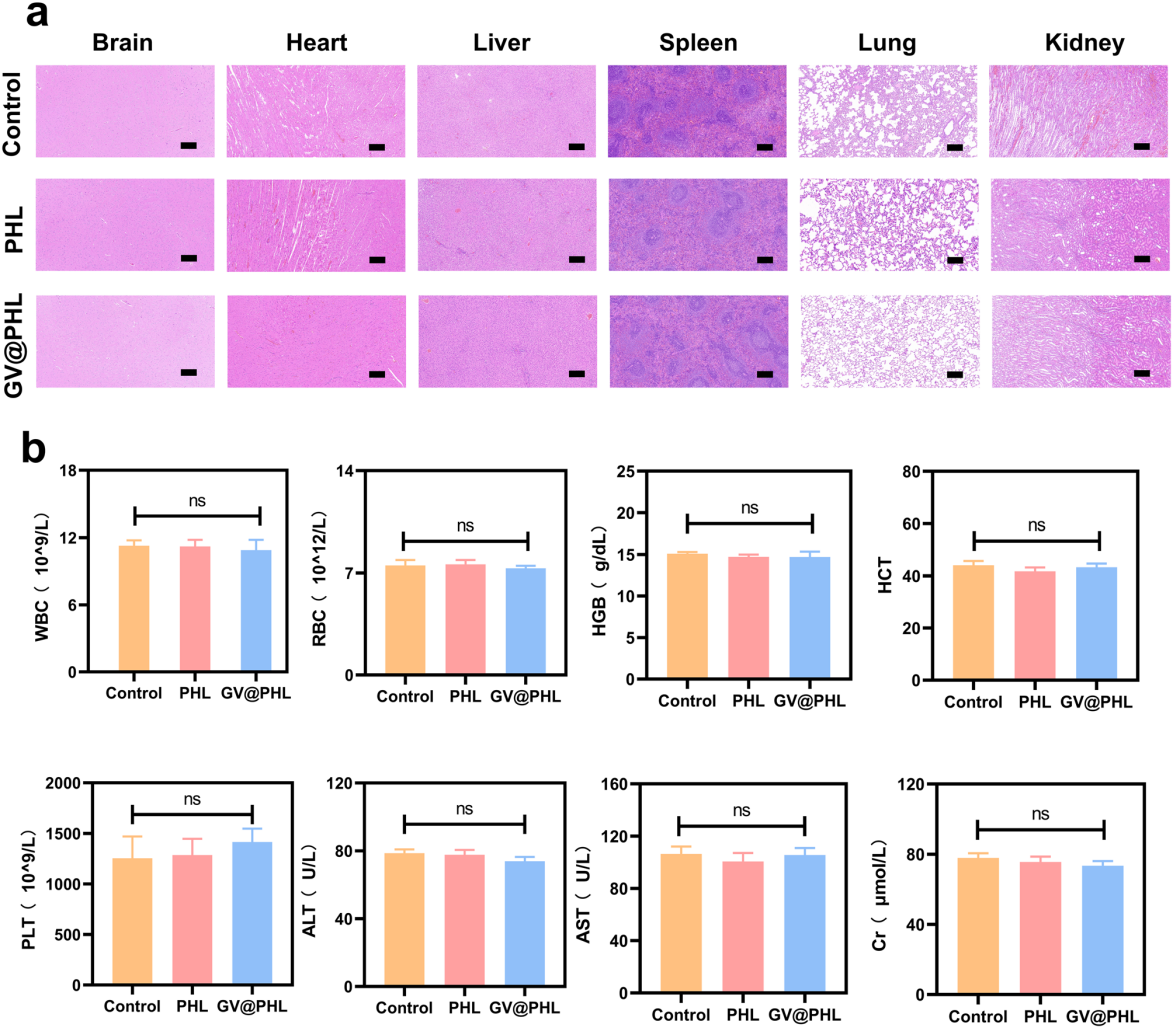
Biosafety experiments of PHL scaffold and GV@PHL Scaffold in vivo. (**a**) HE staining of the major rat organs after treatment for 6 weeks (Scale bar = 200 μm). **(b)** Analysis of blood biochemical parameters in different groups of rats. All experimental data are expressed as mean ± SD (*n* = 3). *p < 0.05, **p < 0.01, ***p < 0.001

Six weeks after scaffold transplantation, histological staining of major organs from SD rats (including the heart, liver, spleen, lungs, and kidneys, key organs for evaluating systemic toxicity) was performed to assess potential adverse responses to scaffold implantation or degradation. As shown in Fig. 7a, H&E staining revealed that all major organs maintained their normal histological architecture: the heart showed intact myocardial fibers without inflammatory cell infiltration or necrosis; the liver exhibited regular hepatic lobule structure and no signs of hepatocellular degeneration or portal tract inflammation; the spleen retained normal white and red pulp distribution, with no abnormal lymphocyte aggregation or tissue fibrosis; the lungs displayed intact alveolar structures and no interstitial edema or inflammatory exudate; and the kidneys showed normal glomerular and tubular morphology, with no tubular necrosis or glomerular congestion. These findings confirmed that scaffold transplantation and subsequent degradation did not elicit systemic inflammatory responses or cause structural damage to rat organs.

To further evaluate the functional integrity of vital organs, we measured serum biochemical markers of liver and kidney function at the 6-week time point (Fig. 7b). For liver function, alanine transaminase (ALT) and aspartate transaminase (AST), key enzymes released into the bloodstream upon hepatocellular injury, showed no significant differences in levels between the GV@PHL group, PHL group, and blank control group (*p* > 0.05). Similarly, serum creatinine (CRE), a classic marker of renal glomerular filtration function, was comparable across all groups, indicating that scaffold implantation did not impair renal excretory function. These biochemical data complemented the histological observations, confirming that the GV@PHL scaffold does not induce obvious functional damage to the liver or kidneys.

We also analyzed peripheral blood hematological parameters to assess potential hematotoxicity or systemic immune activation (Fig. 7b). White blood cell (WBC) counts, an indicator of systemic inflammation or infection, were within the normal physiological range for SD rats in all groups, with no significant elevations or reductions (*p* > 0.05). Red blood cell (RBC) counts, hemoglobin (HGB) concentrations, and hematocrit (HCT), markers of oxygen-carrying capacity and erythropoiesis, were also comparable across groups, ruling out scaffold-induced anemia or erythropoietic suppression. Additionally, platelet (PLT) counts, critical for coagulation, remained within the normal range, indicating that the scaffold does not interfere with hemostatic function.

Based on the above analysis, the histological, biochemical, and hematological data demonstrate that the GV@PHL scaffold exhibits in vivo biosafety. Neither the scaffold itself nor its degradation products induce obvious systemic toxicity, inflammatory responses, organ damage, or hematological abnormalities in SD rats. This safety profile is critical for the clinical translation of the GV@PHL scaffold, as it ensures that the scaffold’s therapeutic benefits (i.e., enhanced bone regeneration) are not offset by adverse side effects.

## Discussion

The regeneration of large bone defects remains a significant clinical challenge due to the need to coordinate osteogenesis with rapid and durable vascularization [47, 48], all within a mechanically stable scaffold. Conventional polymeric scaffolds (e.g., PCL, PLGA, PLA, etc.) provide structural support but are biologically inert; bioactive ceramics (e.g., HA) improve osteoconductivity but are brittle; and pro-angiogenic hydrogels deliver growth factors but lack load-bearing capacity or spatiotemporal control in defect sites. Bridging these competing demands, including printability, mechanical robustness, osteoinductivity, and sustained angiogenic signaling, is a persistent challenge in the fields of craniofacial and orthopedic tissue engineering.

Despite the impressive advancements in 3D-printed composite scaffold designs, there remain areas where further refinements could enhance their efficacy. While many studies have made significant progress in balancing mechanical integrity with biological activity, the challenge of burst release growth factor delivery for stable vascularization persists, often requiring controllability over release profiles [49–52]. Similarly, maintaining architectural fidelity, crucial for consistent cell infiltration and nutrient exchange, continues to be an area of ongoing research focus.

Here, we developed a two-component scaffold system capable of integrating both vascular and osteogenic properties within a printable structure, with results demonstrating its repair effectiveness at the animal level, mechanically robust framework, coupled a covalently tethered, photo-crosslinkable pro-angiogenic hydrogel. First, we formulated a PCL-nano-HA-Laponite (PHL) ink and systemically mapped its printability window by quantifying filament expansion (*α*), interlayer diffusion (*θ*), and vertical sagging (*γ*) across nozzle sizes and nanoclay concentrations. Rheological testing confirmed shear-thinning behavior and solid-like gel properties, supporting extrusion-based 3D printing with high shape fidelity, while SEM/EDS analyses verified the scaffold’s microstructure and composition. Mechanical testing showed the PHL scaffold’s capacity to withstand compressive stress (≈ 6 MPa at 40% strain for 2% Laponite) and maintained fatigue resistance, highlighting its dimensional stability. Biological functionality was confirmed through in vitro assays with mBMSCs. The scaffold demonstrated biocompatibility, promoted cell proliferation, and accelerated migration (≈ 77% wound closure at 12 h versus ~ 20% in controls). Under osteogenic induction, performance was further underscored by the higher ALP and ARS activity levels, likely due to the synergistic effects of HA and Laponite in enhancing osteogenic differentiation. To incorporate angiogenic functionality, GelMA-VEGF hydrogels were synthesized using EDC/NHS coupling, offering ECM-like porosity, substantial VEGF retention with reduced early burst release, and strong angiogenic potential evidenced by enhanced endothelial network formation, RFP-HUVEC sprouting, and lumen-like structures. Embedding this hydrogel into the pores of the PHL scaffold (GV@PHL) enabled a combined vascularized-osteogenic microenvironment. This study presents GelMA-VEGF hydrogel as a promising approach for tissue engineering and regenerative medicine. Although the material demonstrates potential in promoting angiogenesis and tissue repair, we acknowledge that our research is still in the experimental stage, and there is a long way to go before clinical application. The material’s tunable properties suggest it could be adapted for various applications, including bone, cartilage, and nerve regeneration. However, while our results are promising, significant work remains to scale up production and optimize the manufacturing process for clinical use. Furthermore, regulatory challenges must be addressed, and extensive preclinical testing is required to meet safety and efficacy standards. The controlled release of VEGF must also be carefully evaluated to ensure it does not cause undesirable side effects. Angiogenesis is a dynamic process that initiates rapidly after injury/implantation and then evolves through sprouting and remodeling. In the present work, we quantified VEGF cumulative releas within the early 72 h window, which primarily corresponds to the initial signaling phase for endothelial activation and early sprouting. Clear differences were observed among groups. As expected, the GelMA (blank) control showed negligible VEGF signal. The GelMA+VEGF group (physical mixing) displayed a comparatively higher early release (≈ 60% within 72 h), consistent with diffusion-driven loss of unbound VEGF from the hydrogel network. In contrast, the GelMA-VEGF group (covalent conjugation) exhibited a significantly more gradual and uniform release over 0–72 h, indicating that tethering VEGF to the matrix effectively mitigates burst release and helps maintain local retention during the critical early period.

From a physiological perspective, limiting burst release can be beneficial because an excessive initial spike may be followed by rapid depletion. Thus, the early-time kinetics of GelMA-VEGF are consistent with our design goal of providing controlled VEGF availability immediately after implantation, when pro-angiogenic cues are needed to initiate vascular responses. However, because our in vitro release study was limited to 72 h, we cannot directly conclude multi-week relevance from these data alone. Longer-term release measurements and/or degradation-coupled release under more physiologically relevant conditions (e.g., enzymatic degradation) will be important to determine whether sustained VEGF presentation can be maintained over extended periods. From a physiological perspective, limiting burst release can be beneficial because an excessive initial spike may be followed by rapid depletion. Thus, the early-time kinetics of GelMA-VEGF are consistent with our design goal of providing controlled VEGF availability immediately after implantation, when pro-angiogenic cues are needed to initiate vascular responses. However, because our in vitro release study was limited to 72 h, we cannot directly conclude multi-week relevance from these data alone. Longer-term release measurements and/or degradation-coupled release under more physiologically relevant conditions (e.g., enzymatic degradation) will be important to determine whether sustained VEGF presentation can be maintained over extended periods.

In the in vivo evaluation using the rat cranial defect model, the GV@PHL scaffold demonstrated therapeutic effects. Micro CT and histological staining showed that the GV@PHL group achieved almost complete defect closure, accompanied by abundant new blood vessel formation. Hematological assessments and organ tissue staining confirmed the biocompatibility of the system at 6 weeks.These results highlight the GV@PHL system’s ability to promote bone regeneration, vascularization, and biosafety with structural integrity and mechanical stability.

While the results demonstrate the promise of the GV@PHL strategy in promoting vascularized osteogenesis, certain limitations remain that warrant further investigation. The current in vivo evaluation was conducted using a non-load-bearing calvarial defect model with a relatively short follow-up period (6–8 weeks). Although the test results showed no significant scaffold residue, the degradation properties of the scaffold have not been studied. Long-term studies in load-bearing models are necessary to evaluate scaffold degradation kinetics, its mechanical integration with remodeling bone, and the durability of newly formed vasculature under physiological conditions. While the results suggest that VEGF release may play a role in supporting osteogenic pathways and promoting vascular microenvironment formation, these mechanistic effects are inferred from correlative data and require further investigation. The observed synergistic effects between VEGF release and bone regeneration are promising, but more detailed studies are needed to confirm the precise mechanisms underlying these interactions.

Indeed, the integration of structural integrity, osteogenic capacity, into a single platform represents a meaningful advancement, yet the complexities of translating these findings into clinical applications demand ongoing efforts. Addressing these challenges will be critical for advancing the GV@PHL system toward clinical translation in large bone defect regeneration. This work lays a foundational framework that encourages further exploration of the synergistic effects of composite materials within regenerative medicine.

## Supplementary Information

Below is the link to the electronic supplementary material.

Supplementary Material 1

## Data Availability

The authors declared that all the Data in this work is available on the request from the corresponding author (Z., L: zhangl@lpbr.cn).
