# Supplementary material for: Engineering a vascularized-osteogenic microenvironment to enhance bone regeneration via a 3D-printed composite scaffold with progressive-release bio-factors
Source: J Transl Med. 2026 Apr 10;24:522. doi: 10.1186/s12967-026-08090-5 (PMC13088540; doi:10.1186/s12967-026-08090-5)
Supplement: Supplementary file 1 — Supplementary Material 1 [file 12967_2026_8090_MOESM1_ESM.docx]

**Fig.S1** Quantitative analysis of ALP activity by ALP detecting kit

**Fig. S2 Cumulative VEGF release (%)**

| PHA | PCL+nHA |
| --- | --- |
| PHL | PCL+nHA+2%Lapnoite |
| GelMA-VEGF(GV) | Covalent binding of GelMA with VEGF |
| GelMA+VEGF | Physical mixing of GelMA with VEGF |
| GV@PHL | Composite bone-forming and angiogenesis-promoting scaffold |

**Table S1： Illustration of Abbreviations Used in This Work**
